# Changes in Temporal Properties of Notifiable Infectious Disease Epidemics in China During the COVID-19 Pandemic: Population-Based Surveillance Study

**DOI:** 10.2196/35343

**Published:** 2022-06-23

**Authors:** Xixi Zhao, Meijia Li, Naem Haihambo, Jianhua Jin, Yimeng Zeng, Jinyi Qiu, Mingrou Guo, Yuyao Zhu, Zhirui Li, Jiaxin Liu, Jiayi Teng, Sixiao Li, Ya-nan Zhao, Yanxiang Cao, Xuemei Wang, Yaqiong Li, Michel Gao, Xiaoyang Feng, Chuanliang Han

**Affiliations:** 1 The National Clinical Research Center for Mental Disorders Beijing Anding Hospital Capital Medical University Beijing China; 2 Advanced Innovation Center for Human Brain Protection Capital Medical University Beijing China; 3 Beijing Key Laboratory of Mental Disorders Beijing Anding Hospital Capital Medical University Beijing China; 4 Faculty of Psychology and Center for Neuroscience Vrije Universiteit Brussel Brussel Belgium; 5 Zhongshan School of Medicine Sun Yat-sen University Guangzhou China; 6 State Key Laboratory of Cognitive Neuroscience and Learning Beijing Normal Univeristy Beijing China; 7 IDG/McGovern Institute for Brain Research Beijing Normal Univeristy Beijing China; 8 School of Artificial Intelligence Beijing Normal University Beijing China; 9 Brain Cognition and Brain Disease Institute Shenzhen Institute of Advanced Technology Chinese Academy of Sciences Shenzhen China; 10 Shenzhen Key Laboratory of Neuropsychiatric Modulation and Collaborative Innovation Center for Brain Science Guangdong Provincial Key Laboratory of Brain Connectome and Behavior Chinese Academy of Sciences Shenzhen China; 11 College of Environmental Sciences and Engineering Peking University Beijing China; 12 Baoding First Central Hospital Baoding China; 13 Department of Psychology University of Washington, Seattle Seattle, WA United States; 14 School of Psychology, Philosophy and Language Science University of Edinburgh Edinburgh United Kingdom; 15 School of Music Faculty of Arts University of Leeds Leeds United Kingdom; 16 Institute of Acupuncture and Moxibustion China Academy of Chinese Medical Sciences Beijing China; 17 WM Therapeutics Ltd Beijing China; 18 Institute of Mental Health Peking University Sixth Hospital Beijing China

**Keywords:** class B infectious disease, COVID-19, event-related trough, infection selectivity, oscillation, public health interventions, pandemic, surveillance, health policy, epidemiology, prevention policy, public health, risk prevention

## Abstract

**Background:**

COVID-19 was first reported in 2019, and the Chinese government immediately carried out stringent and effective control measures in response to the epidemic.

**Objective:**

Nonpharmaceutical interventions (NPIs) may have impacted incidences of other infectious diseases as well. Potential explanations underlying this reduction, however, are not clear. Hence, in this study, we aim to study the influence of the COVID-19 prevention policies on other infectious diseases (mainly class B infectious diseases) in China.

**Methods:**

Time series data sets between 2017 and 2021 for 23 notifiable infectious diseases were extracted from public data sets from the National Health Commission of the People’s Republic of China. Several indices (peak and trough amplitudes, infection selectivity, preferred time to outbreak, oscillatory strength) of each infectious disease were calculated before and after the COVID-19 outbreak.

**Results:**

We found that the prevention and control policies for COVID-19 had a strong, significant reduction effect on outbreaks of other infectious diseases. A clear event-related trough (ERT) was observed after the outbreak of COVID-19 under the strict control policies, and its decreasing amplitude is related to the infection selectivity and preferred outbreak time of the disease before COVID-19. We also calculated the oscillatory strength before and after the COVID-19 outbreak and found that it was significantly stronger before the COVID-19 outbreak and does not correlate with the trough amplitude.

**Conclusions:**

Our results directly demonstrate that prevention policies for COVID-19 have immediate additional benefits for controlling most class B infectious diseases, and several factors (infection selectivity, preferred outbreak time) may have contributed to the reduction in outbreaks. This study may guide the implementation of nonpharmaceutical interventions to control a wider range of infectious diseases.

## Introduction

Atypical pneumonia caused by a new coronavirus was first reported in December 2019 [1-4] and was subsequently termed “COVID-19” by the World Health Organization (WHO) on February 12, 2020. Later, human-to-human transmission of COVID-19 was confirmed, resulting in a pandemic outbreak worldwide [5-13]. After the outbreak, the Chinese government took immediate action to implement strict public health policies [14], such as lockdown, quarantine measures, and social distancing. Domestic and international travel was restricted, mass gatherings were reduced, and public entertainment venues and schools were closed. The government also asked people to be more vigilant and take personal precautions, such as sanitizing hands and wearing surgical masks. Under these policies, the number of COVID-19 infections in China sharply decreased, and this situation has lasted until recently [15-17]. In addition to the COVID-19 outbreak, other fatal infectious diseases have also had outbreaks [18], which may have been overlooked. In China, the national infectious disease surveillance system has been recording outbreaks of other diseases [19]. Infectious diseases are divided into notifiable classes A, B, and C. In this classification, class B notifiable diseases have the potential to cause severe epidemic outbreaks, such as hepatitis B virus (HBV) [20], scarlet fever [21], measles [22], and rabies [23-25]. Notably, COVID-19 is classified as a class B disease.

During the COVID-19 pandemic, local and international governments relied on nonpharmaceutical measures until vaccines were available. Unlike vaccines or medicine, which are restricted by supply and logistics [26], nonpharmaceutical interventions (NPIs) could have a broader impact on multiple infectious diseases. Take the influenza virus as an example. Human beings have little immunity to it, which allows it to spread rapidly from one person to another. In the absence of effective vaccines to immunize people, NPIs are one of the best strategies to control pandemics. Several studies have found that policies to prevent COVID-19 and other NPIs could reduce the number of infections of influenza [17,27-29], tuberculosis [30,31], and some other diseases [32,33] to a large degree, while the characteristics of an epidemic are not only limited to the static number of the infected cases but also limited to the temporal dynamics of the epidemic. The temporal features of an infectious epidemic after NPIs is not precisely defined, although common sense suggests that the number of cases may decrease. The question whether under a consistent and rigorous prevention policy, this decrease would rebound or only fall to 0 arises. New analysis indicators are required to define it clearly. There are some characteristics of temporal dynamics, such as the tuning curve of the infectious disease in a year [34-37] and the spectrogram of the epidemic [38-42] analyzed by the Fourier method. The tuning curve of monthly infected cases illustrates the essential profile of each disease outbreak and gives a direct picture of the monthly situation, but it lacks quantitative features (eg, infection selectivity and preferred outbreak time) that were highly summarized from the tuning curve and lack of further analysis. Although these temporal indices have been mentioned in previous studies, it remains unclear how they changed with strict NPIs during the COVID-19 outbreak and to what extent they contributed to the reduction in infectious cases under the NPIs.

In the light of this, in this study, we investigated the impact of NPIs on other class B infectious diseases. We extracted the time series data for 23 class B notifiable infectious diseases between 2017 to 2021 from public data sets of the National Health Commission of the People’s Republic of China [43]. During the COVID-19 pandemic, the strict NPIs in China have always been existing, which can be described by the stringency index taken from the Oxford COVID-19 Government Response Tracker [44]. We expected to find a significant trough of most class B infectious diseases after the outbreak and subsequent interventions for COVID-19, which we defined as the event-related trough (ERT). The ERT can be used to investigate the fluctuations in several infections that are time-locked to an event without intervention. We then explored how infection selectivity and the preferred month of the outbreak of the infectious diseases may affect the ERT. Finally, we calculated the oscillatory strength of each infectious disease and compared the power before and after the COVID-19 outbreak.

## Methods

### Data and Sources

Time series data available for the monthly reported and confirmed cases of 23 class B notifiable infectious diseases in China’s mainland, from April 2017 to September 2021, were obtained from the National Health Commission of the People’s Republic of China. The data set is open to the public around the world and is reported by the Chinese Centre for Disease Control and Prevention (CDC) each month. These 23 diseases are HIV/AIDS, hepatitis (including hepatitis A virus, HAV; HBV; hepatitis C virus, HCV; and hepatitis E virus, HEV), measles, hemorrhagic fever, dengue and severe dengue, rabies, Japanese encephalitis, anthrax, *Shigella* spp. or *Entamoeba histolytica*, tuberculosis, typhoid and paratyphoid fever, pertussis, neonatal tetanus, scarlet fever, brucellosis, gonorrhea, *Treponema pallidum*, leptospirosis, schistosomiasis, and malaria. The data sampling rate was 1 time point per month (12 time points per year) from the monthly report of the National Health Commission of the People’s Republic of China. We used 2 criteria to select these diseases. First, the maximum number of infectious cases each month in recent years should be larger than 10. Second, the time points should be continuously publicly reported within the years of interest. We were mainly interested in how other class B infectious diseases might be influenced by policies related to COVID-19, considering that COVID-19 is also classified as class B. We did not include class A diseases due to their low incidences. Class C diseases, such as the flu, were not included, because they are less fatal and controllable and would not have the same impact as class B diseases.

Indicators of government response in China were taken from the Oxford COVID-19 Government Response Tracker [44]. In this work, we use the stringency index (all closure indicators, such as lockdown policies and travel bans, and health system policies that record public information campaigns and contact tracing), which records the strictness of lockdown-style policies. The index scores the level of government responses between 0 and 100. The higher the score is, the stricter the government interventions were (Multimedia Appendix 1).

### Ethical Considerations

For this study, we used public data from the National Health Commission of the People’s Republic of China. Our study did not involve any intervention on human participants. This study was approved by the Ethics Committee of Beijing Anding Hospital, Capital Medical University, China.

### Trough and Peak Amplitude Before and After the COVID-19 Outbreak

We defined a new concept named the ERT, which originates from the event-related potential (ERP) in neuroscience [45]. The ERT describes the direct impact of specific events on reducing the number of infectious diseases. This event could be a pharmaceutical or nonpharmaceutical intervention to prevent the spread of infectious disease. In this study, the specific event is the strong prevention and control policies implemented at the outbreak of the COVID-19 epidemic, which are an NPI. The ERT is the lowest increase of an outbreak in the period of 6 months after the outbreak of COVID-19. The trough amplitude before COVID-19 is the lowest value of the infection in the 3-year period before COVID-19 (Equation 1). The peak amplitude (Equation 2) before and after the COVID-19 outbreak is the highest value of the infection before and after COVID-19. We also calculated the trough ratio index as the ratio of troughs before and after the outbreak of COVID-19 (Equation 3).

Trough amplitude = arg min(infected cases after outbreak of the epidemic) (1)

Peak amplitude = arg max(infected cases after outbreak of the epidemic) (2)

Trough ratio index = arg min(infected cases before outbreak of the epidemic)/arg min(infected cases after outbreak of the epidemic) (3)

### Tuning Curves for Monthly Infected Cases Before and After the COVID-19 Outbreak

The tuning curve of the monthly infected cases illustrates the essential profile of the outbreak of each disease in China, which gives a direct picture of the situation each month based on the historical data. We assumed that all infectious diseases included in this study have a similar trend each year for the years of observation (Multimedia Appendix 2), similar to previous studies [18]. Thus, we took the monthly average number of infected cases and computed them into a tuning curve (Equation 4). Each infectious disease in this study has a tuning curve before and after the COVID-19 outbreak, and the oscillatory pattern within a year is clear.

Tuning curve_month_ = sum(infected cases_month_)/N, (4)

where N is the number of years.

### Preferred Month and Selectivity of the Epidemic Outbreak Before and After COVID-19

Two indices of the disease were defined: preferred month and infection selectivity (Equation 5), which are important indicators of the infectious property of the epidemics caused by a disease in a year. The preferred month index is defined as the month in a year that has the most cases of infections. The infection selectivity index is defined as (1 – ratio of the minimum and the maximum number of infected cases in a year). If the selectivity index is closer to 1, it means outbreaks only occur in specific months. If the selectivity index is closer to 0, it means that outbreaks occur throughout the year.

Selectivity index = 1 – [min(mean infected cases in a year)/max(mean infected cases in a year)] (5)

### Power Spectrum Analysis

The oscillatory property of an infectious disease is an important indicator of the regular fluctuations and recurrence of epidemics. To better quantify these fluctuations, we used spectrum analysis. Similar methods have been used in classic and modern studies in the field of infectious diseases [38-42] and some other biological research [46,47]. Spectrum analysis is a technique for decomposing complex signals into simpler signals based on Fourier transform (Equation 6). Many biological signals can be expressed as the sum of various simple signals of different frequencies and produce information about a signal at different frequencies (eg, amplitude, power, intensity, phase).



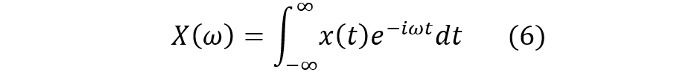



The power spectral density (PSD; Equation 7) for each infectious disease before and after the outbreak of COVID-19 was computed using the multitaper method with the Chronux toolbox [48], an open source, data analysis toolbox [49]. Power spectra of the time series data (infected cases of each disease) were calculated in 2 time periods (2017-2020 and 2020-2021).



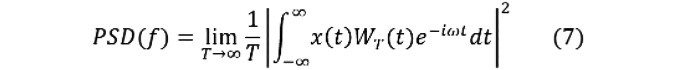



where W_T_(t) is 1 within the arbitrary period and 0 elsewhere, and T is centered about some arbitrary time t=t_0_.

### Correlation Analysis

We performed Pearson correlation to measure the relationship of several indices (ERT, selectivity, oscillatory strength, and mean infected number) before and after the COVID-19 outbreak. Pearson correlation was also performed in the correlation analysis between trough ratio and selectivity, between the change in power and change in infected numbers, and between the change in power and change in trough amplitude. Spearman correlation was performed to measure the relationship between the trough amplitude and the peak amplitude before and after the COVID-19 outbreak. The significance (*P* value) of the correlation was corrected with Bonferroni correction.

### Statistical Analysis

We performed an independent-sample *t* test to compare the difference between several indices (trough amplitude, peak amplitude) before and after the COVID-19 outbreak and test the difference in the trough ratio between diseases with a different preferred time of outbreak. The pairwise *t* test was performed to compare the oscillatory power and the average infected number before and after the COVID-19 outbreak.

## Results

### Monthly Data

**Figure 1:**
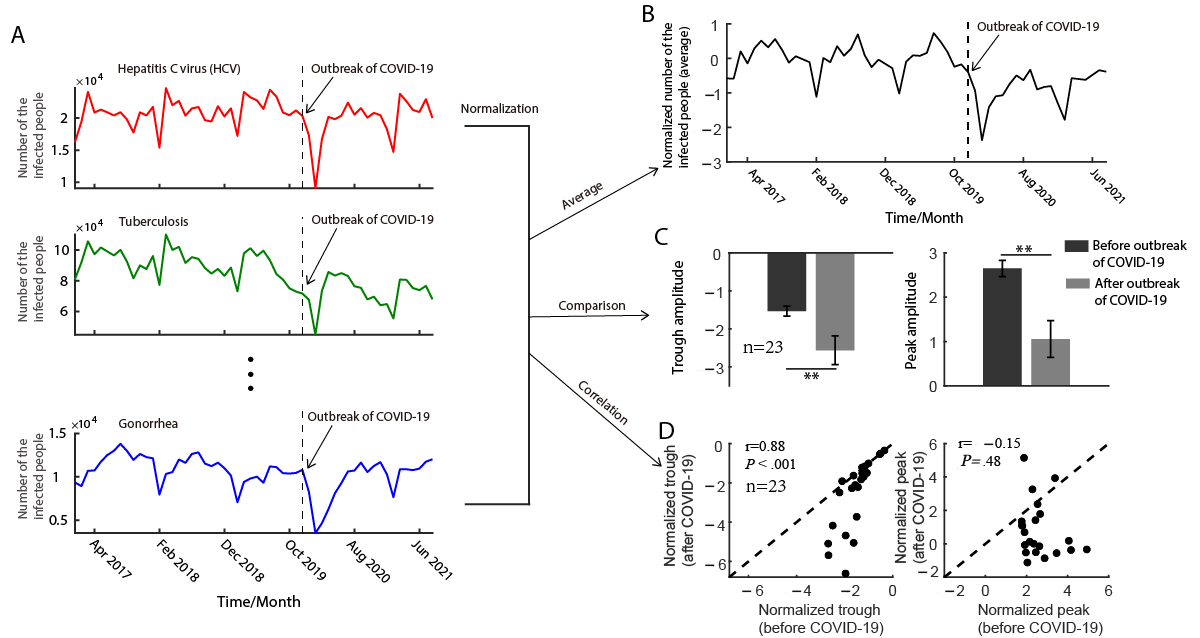
Infectious disease before and after the COVID-19 outbreak (A) Monthly infected cases from 2017 to 2021 of three examples (HCV, Tuberculosis, and Gonorrhea). The curve after the vertical dotted line shows specifically the infected cases after the COVID-19 outbreak. (B) The normalized mean number of infected cases before and after the COVID-19 outbreak. (C) Trough amplitude (left) and peak amplitude (right) before and after the COVID-19 outbreak (** is for *P*<.01). (D) The relationship between the normalized trough (left) and peak (right) before and after the COVID-19 outbreak.

This study analyzed monthly data from April 2017 to September 2021 of confirmed cases of 23 class B notifiable infectious diseases in China’s mainland. After the COVID-19 outbreak, most class B infectious diseases had an obvious sudden trough, which we defined as the ERT (see Figure 1A for 3 typical examples). The stringency index of China showed that during the COVID-19 pandemic, the strict NPIs in China have always been existing (Multimedia Appendix 1), which allows us to analyze the long-term effect after the COVID-19 outbreak.

### Significant Event-Related Trough Occurred After the Strict Control Policy for the COVID-19 Outbreak in China

We showed several examples of diseases that had an obvious ERT after the COVID-19 outbreak (HCV, tuberculosis, and gonorrhea); see Figure 1A, and see Multimedia Appendix 3 for all diseases. To compare the time series of all diseases on a notionally common scale, we normalized the time series of the number of infected people by subtracting the mean number of infections before COVID-19 and dividing it by its SD. Hence, the mean number of infections before COVID-19 was 0 for all the diseases (Figure 1B). The pattern shows an obvious and sudden decrease in confirmed cases after the COVID-19 outbreak (see Figure 1B). To investigate whether the peak and trough amplitudes may change due to the outbreak, we compared the differences between peak and trough amplitudes before and after the event (policy in response to COVID-19). Results showed that the amplitude of both peak (*P*<.01) and trough (*P*<.01) significantly decreased, which indicated that the outbreak strongly moderated the oscillation amplitude (see Figure 1C). We then calculated the correlation between the normalized trough before and after the outbreak event, and we found that the trough after the outbreak was significantly correlated (r=0.88, *P*<.001) to the trough before but the peak was not correlated (r=–0.15, *P*=.48; see Figure 1D).

### Infection Selectivity and Preferred Outbreak Time Strongly Related to the Trough Ratio Before and After the COVID-19 Outbreak

**Figure 2:**
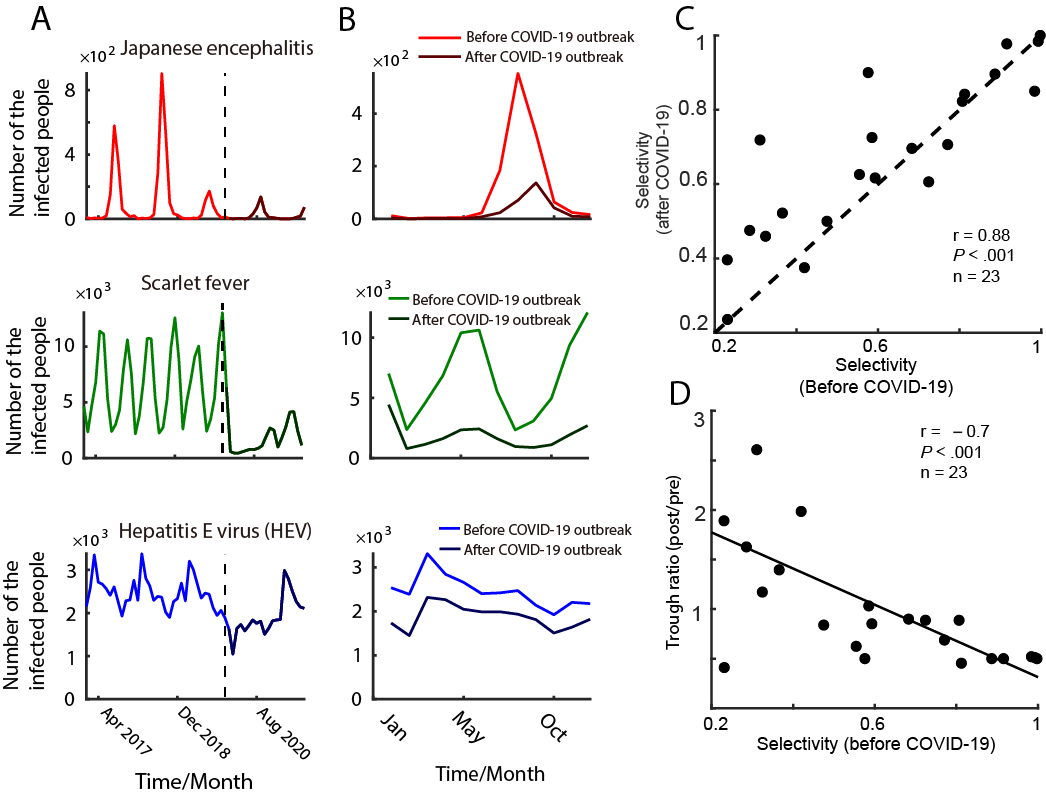
Relationship between selectivity and trough ratio before and after the COVID-19 outbreak (A) Monthly infected cases from 2017 to 2021 of three examples (Japanese encephalitis, Scarlet fever, and HEV). The curve after the vertical dotted line shows specifically the infected cases after the COVID-19 outbreak. (B) The number of infected cases every month in a year calculated before (light-colored curve) and after the COVID-19 outbreak (dark-colored curve) corresponding to the time-series data of plot A. (C) The scatter plot of the selectivity before and after the COVID-19 outbreak. (D) The relationship between the selectivity (before) and trough ratio (post/pre).

The ERT might be affected by the basic properties (infection selectivity and preferred outbreak time) of infectious disease outbreaks. To further clarify potential factors that would cause an ERT, we determined the property of oscillations for infectious diseases in a year by defining 2 indicators: infection selectivity and preferred outbreak time of the infectious disease. We selected 3 infectious diseases that have different selectivity as examples (Figure 2A,B; see Multimedia Appendix 4 for all diseases). The infection selectivity index is defined as (1 – ratio of the minimum and the maximum number of infected cases in a year). If the selectivity index is closer to 1, then the shape of the tuning curve is sharper (eg, Japanese encephalitis), and vice versa (eg, HEV). The preferred month index is defined as the month in a year that has the most cases of infections. Results showed that there was a significant increase in infection selectivity after the outbreak of COVID-19, and infection selectivity before and after the outbreak was positively correlated (with Bonferroni correction; Figure 2C). When we compared the selectivity before the outbreak and the trough ratio, we found that the stronger the infection selectivity, the smaller the trough ratio (Figure 2D). We also conducted a partial correlation analysis between infection selectivity and trough ratio, controlling for the preferred time of outbreak, which was significant (r=–0.58, *P*=.004). The association between infection selectivity and trough ratio confounded by seasons was, however, weak.

### Relationship Between an Infection and Its Oscillatory Power Before and After the COVID-19 Outbreak

**Figure 3:**
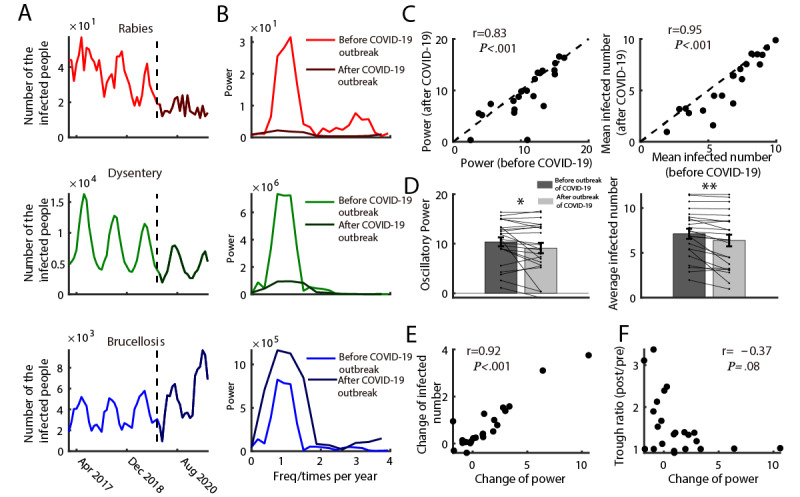
Relationship between the infection and its oscillatory power before and after COVID outbreak (A) Infected cases from 2017 to 2021 of three examples (Rabies, Dysentery, and Brucellosis). The curve after the vertical dotted line represents specifically the infected cases after the COVID-19 outbreak. (B) The power spectrum calculated before (light-colored curve) and after the COVID-19 outbreak (dark-colored curve) corresponding to the time-series data of plot A. (C) The scatter plot of the power (left) and mean infected number (right) before and after the COVID-19 outbreak. (D) The histogram of the oscillatory power (left) and averaged infected cases (right) before and after the COVID-19 outbreak. (E) The scatter plot of the change of power and change of infected number. (F) The scatter plot of the change of power and change of trough amplitude.

We quantified the oscillatory strength of outbreaks using power spectrum analysis (Figures 3A and 3B; see Multimedia Appendix 5 for all diseases). We then explored the relationship between infected cases and their corresponding strength of oscillatory power before and after the COVID-19 outbreak. Results indicated that the oscillatory strength (r=0.83, *P*<.001) and mean infected cases (r=0.95, *P*<.001) before the COVID-19 outbreak were significantly positively correlated to the indices after the COVID-19 outbreak (Figure 3C), showing that the stronger the oscillatory power was before the outbreak, the stronger it was after the outbreak. The same was true for mean infected cases.

To determine the differences between oscillatory power before and after the COVID-19 outbreak and between mean infected cases before and after the COVID-19 outbreak, we also split the data and compared the indices before and after the event. Consistent with our hypothesis, results showed both decreases in oscillatory power and mean infected cases after the COVID-19 outbreak (Figure 3D). We further examined the relationship between the change in power between the change in infected numbers and trough amplitude. Results showed that the change in power and the change in infected numbers was significantly correlated (r=0.92, *P*<.001). The more the change in oscillatory power, the more changes in the number of confirmed cases (Figure 3E). However, the change in power was not related to the change in trough amplitude (r=–0.37, *P*=.08 with Bonferroni correction; Figure 3F). In sum, the COVID-19 outbreak reduced the outbreaks of class B notifiable infectious diseases, as indicated by oscillatory power and mean infected cases.

## Discussion

### Principal Findings

**Figure 4:**
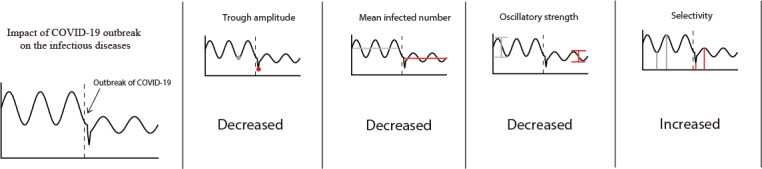
A summary of the main finding. As illustrated in the four plots, the impact of the COVID-outbreak influenced the infectious diseases in four aspects: decreased the trough amplitude, the mean infected cases, and the oscillatory strength, but increased the seasonal selectivity.

In this study, we defined several novel concepts and robust metrics (ERT, selectivity of infection, preferred time to outbreak, oscillatory strength of the infectious disease) to quantify and capture the temporal characteristics of infectious disease outbreaks and event-related fluctuations in China. Our results showed that a clear ERT occurred for most class B infectious diseases after the COVID-19 outbreak under the strict public health policy. We further found that the ERT was related to the nature of diseases, such as their infection selectivity and preferred outbreak time. However, their oscillatory strength was somehow unrelated. We also compared these indices of the infectious diseases before and after the outbreak of COVID-19. The impact of the COVID-19 outbreak influenced the infectious diseases by reducing the trough amplitude, mean infected cases, and oscillatory strength but increasing infectious selectivity (see Figure 4).

### Comparison With Prior Work

To the best of our knowledge, this is the first study to systematically investigate the influence of the COVID-19 outbreak on the temporal characteristics of other class B infectious diseases in China, including both respiratory infectious diseases and other types, such as those transmitted through sex, body fluids, the digestive tract, contact, and mosquitos. The key contribution of this study is that several new concepts were purported, such as the ERT, selectivity of infectious diseases, the preferred outbreak time, and the power strength of infectious oscillation. Some previous studies have investigated basic properties of a few infectious diseases in China [18,22-25,50-52] and countries worldwide [39,40,53-55], and NPIs to mitigate COVID-19 could have affected the transmission dynamics of influenza and other respiratory diseases [17,27-29,56,57]. However, previous research did not quantify the reduction using a specific temporal index. We first defined the ERT in the field of infectious diseases to capture the immediate influence of the strong interventions related to the public health events, such as the prevention policy on disease outbreaks. The ERT could measure the temporal feature when studying the effect of some specific interventions in the future, which increases 1 dimension (temporal dynamics) compared with a simple reduction number.

Another novel finding of this study is that we also built up a connection between the ERT and some other important indicators (selective property and oscillatory property), which were neglected in prior works. In this study, we found that the ERT is related to the selectivity (Figure 2D) of an infectious disease, which gives a new understanding of how an epidemic could be more easily controlled (when a disease has high selectivity). In the future, infectious selectivity would play a more important role than before, especially when combined with the tuning curve of a disease. It would depict new pictures of the basic property of each disease and give more practical guidance on the prevention and control of epidemics. The oscillatory properties of infectious diseases were also analyzed in some previous studies [18,22-25,38-40,53-55,58-60], which could be driven by both natural [11,61,62] and human [63-68] factors. However, prior studies did not investigate the influence of COVID-19 measures or other NPIs to control epidemics on the oscillatory strength of infectious diseases systematically. Our results indicate that the oscillatory strength significantly decreased after the COVID-19 outbreak, which was accompanied by a decrease in the mean infections. This finding supports the conceptual hybrid model [18]. We also found that the oscillatory strength before the COVID-19 outbreak did not correlate to the change in trough, which further suggests that the ERT is not related to some seasonal factors but more to the measurement of the COVID-19 outbreak. The oscillatory phenomenon of population-based epidemics would be the new impetus for the study of public health. In the future, this index could be connected to more natural and human factors, which would contribute to constructing a more generic stimulated model to explain history and predict the future situation.

### Limitations

One limitation of our study is that the data we used are from the entire mainland China but are not specific to different provinces or cities, which may lack spatial resolution. Another limitation of our study is that the results were based on a macroscopic rather than a microscopic view of most class B infectious diseases. Further studies are needed to clarify the deeper underlying mechanisms of the COVID-19 pandemic. With these findings, we could better provide the government with recommendations on the optimal timing to intervene before achieving herd immunity, thereby helping to design fit-for-purpose policies.

### Conclusion

In sum, the study developed a new and potentially universal approach to revealing the dynamics of infectious diseases. The transmissibility and severity of infectious diseases fluctuate regularly. The introduction of the concept of the ERT in infectious diseases can better capture the immediate influence of interventions related to previous public health events. Our results confirmed that early commencement of strong public health interventions has additional benefits on other infectious diseases.

## Appendix

Multimedia Appendix 1 Stringency index in China after January 2020 (data from the Oxford COVID-19 Government Response Tracker).

Multimedia Appendix 2 Annual average of all infected cases diseases. HIV: Human Immunodeficiency Virus; HAV: hepatitis A virus; HBV: hepatitis B virus; HCV: hepatitis C virus; HEV: hepatitis E virus.

Multimedia Appendix 3 Monthly cases of infection for all diseases. HIV: Human Immunodeficiency Virus; HAV: hepatitis A virus; HBV: hepatitis B virus; HCV: hepatitis C virus; HEV: hepatitis E virus.

Multimedia Appendix 4 Annual average of all infected cases of diseases before (gray) and after (black) the COVID-19 outbreak. HIV: Human Immunodeficiency Virus; HAV: hepatitis A virus; HBV: hepatitis B virus; HCV: hepatitis C virus; HEV: hepatitis E virus.

Multimedia Appendix 5 Power spectrum of all infected cases before (gray) and after (black) the COVID-19 outbreak. HIV: Human Immunodeficiency Virus; HAV: hepatitis A virus; HBV: hepatitis B virus; HCV: hepatitis C virus; HEV: hepatitis E virus.

## Abbreviations

ERT: event-related trough
HBV: hepatitis B virus
HCV: hepatitis C virus
HEV: hepatitis E virus
NPI: nonpharmaceutical intervention
