# Supplementary figures and images for: Changes in Temporal Properties of Notifiable Infectious Disease Epidemics in China During the COVID-19 Pandemic: Population-Based Surveillance Study
Source: JMIR Public Health Surveill. 2022 Jun 23;8(6):e35343. doi: 10.2196/35343 (PMC9231598; doi:10.2196/35343)

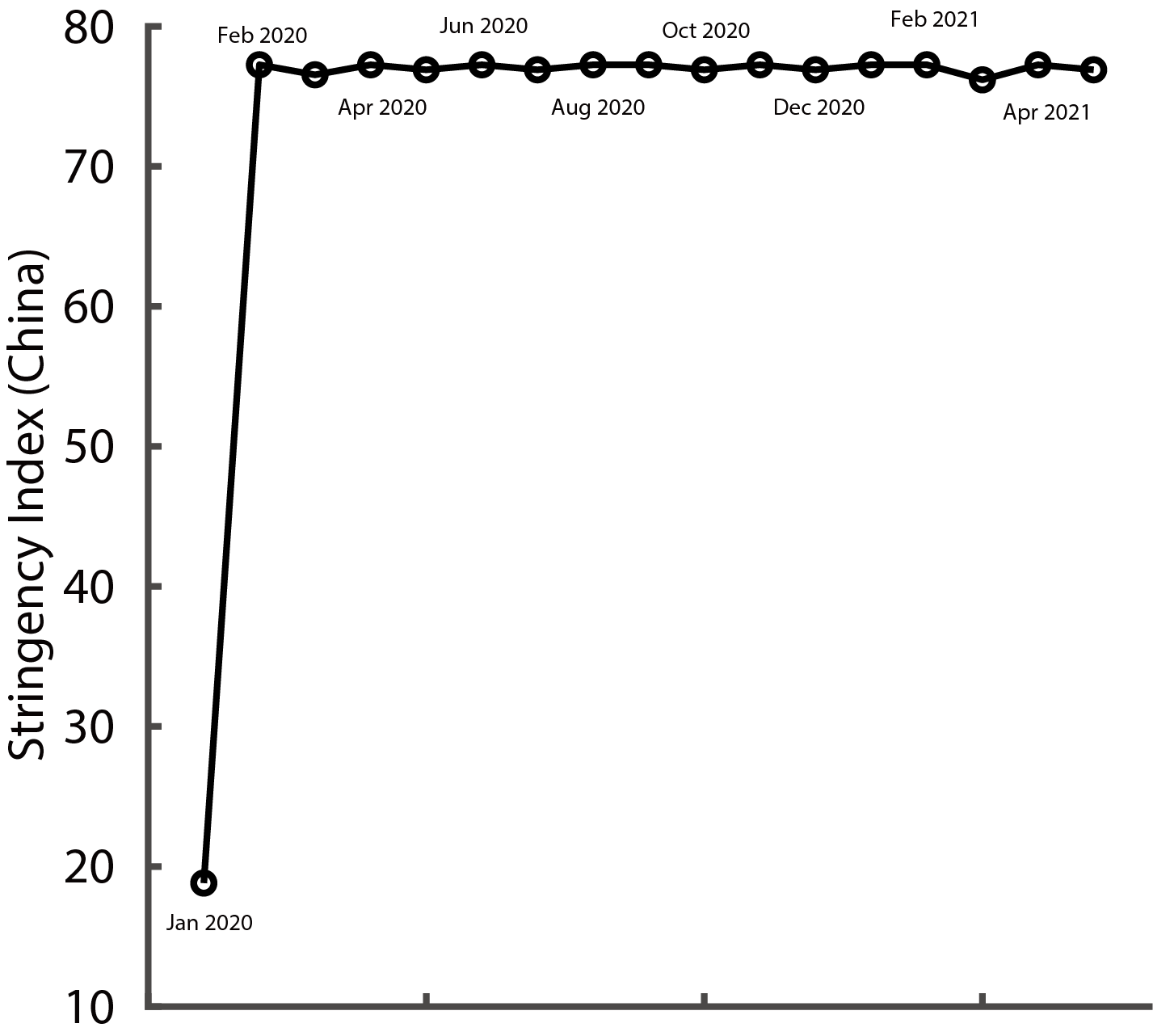

Supplement: Multimedia Appendix 1 [file publichealth_v8i6e35343_app1.png]

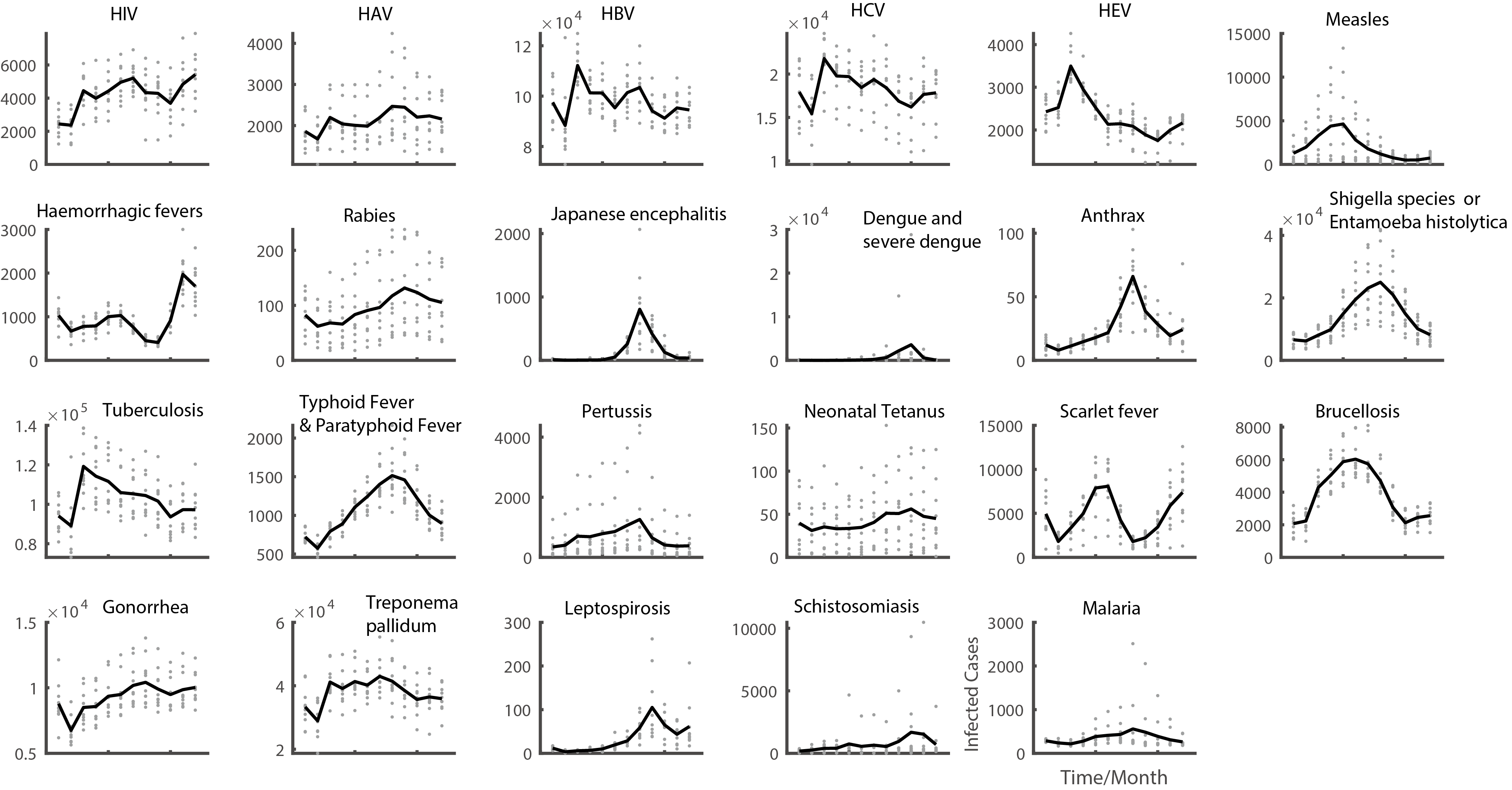

Supplement: Multimedia Appendix 2 [file publichealth_v8i6e35343_app2.png]

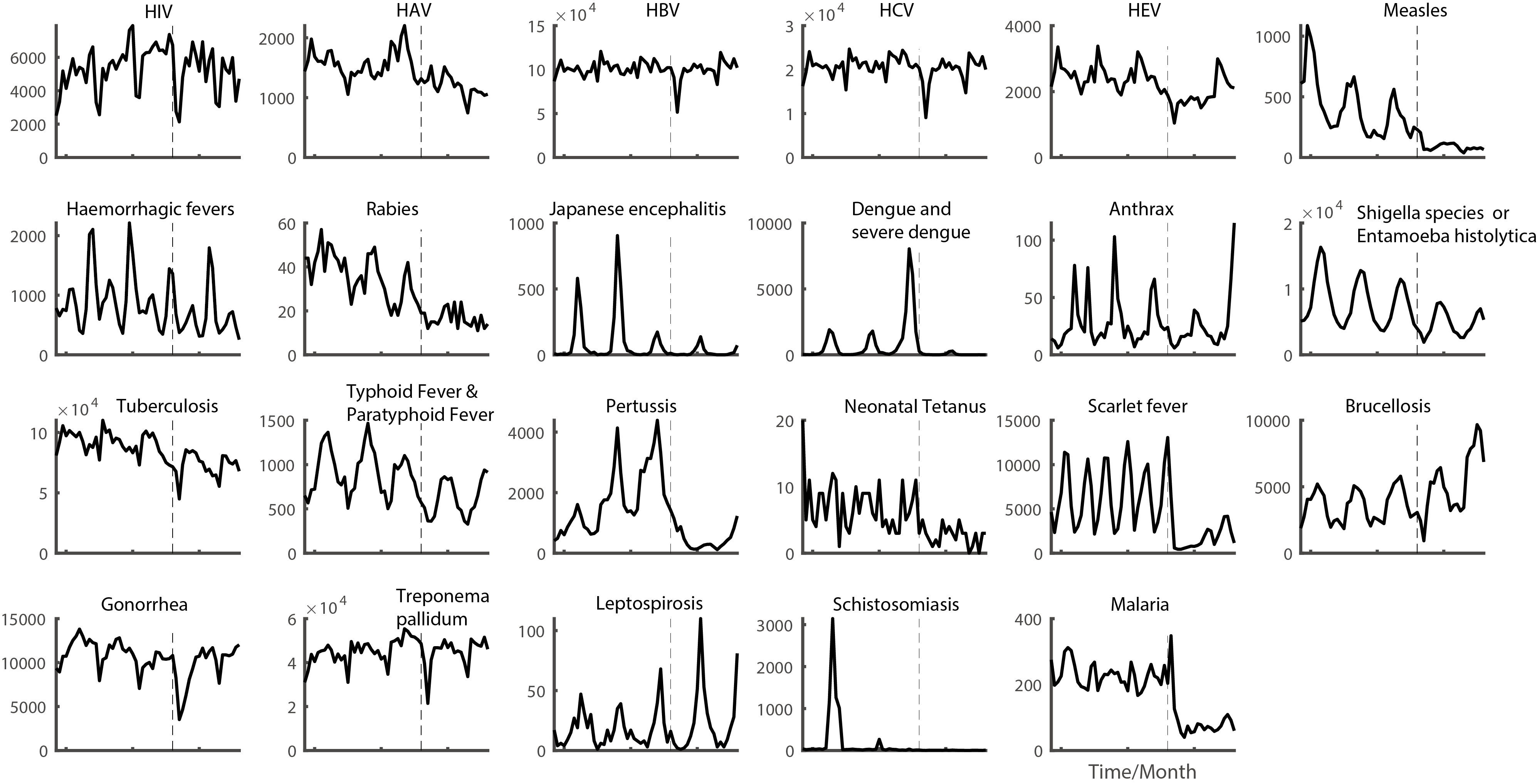

Supplement: Multimedia Appendix 3 [file publichealth_v8i6e35343_app3.png]

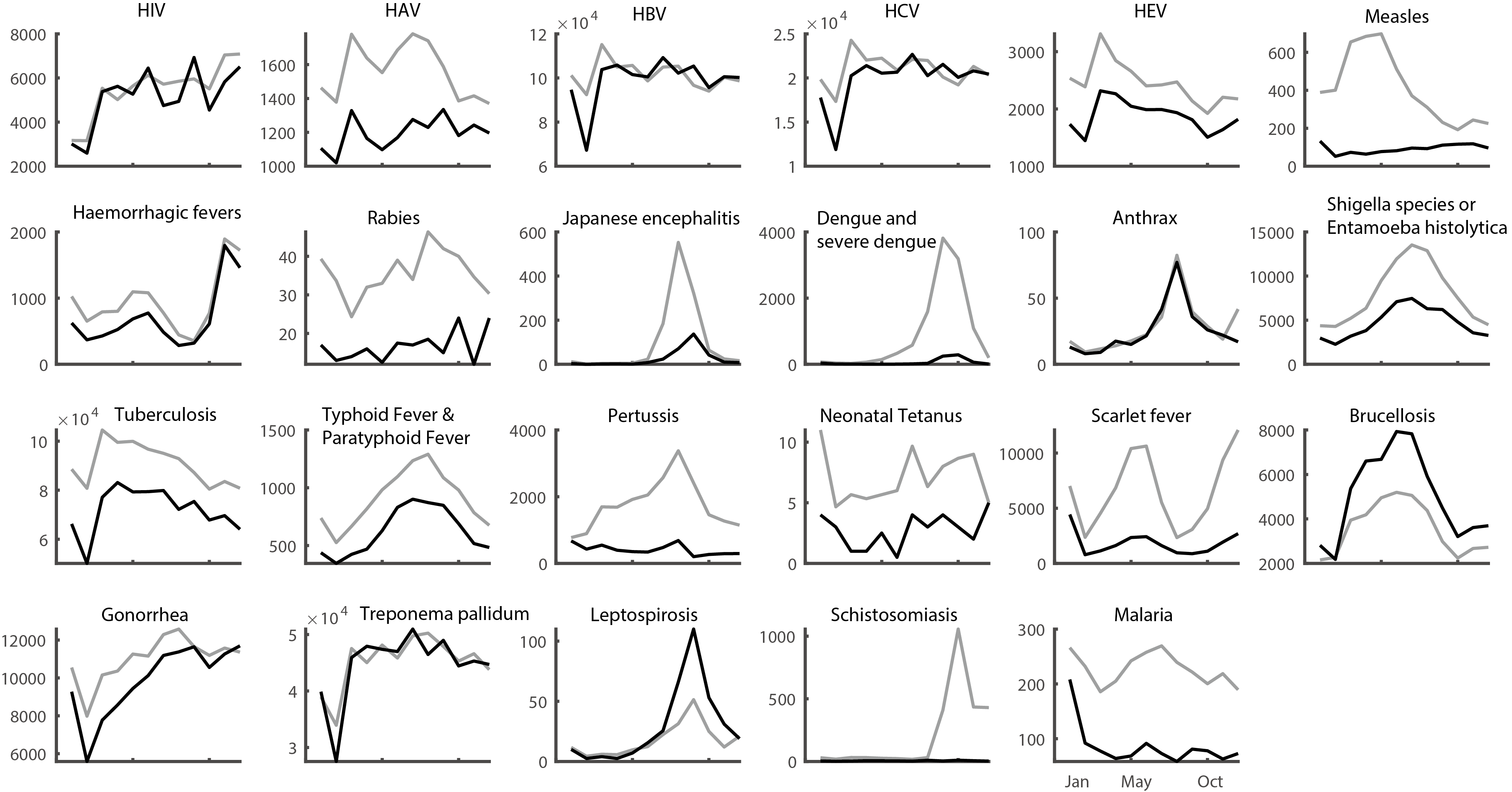

Supplement: Multimedia Appendix 4 [file publichealth_v8i6e35343_app4.png]

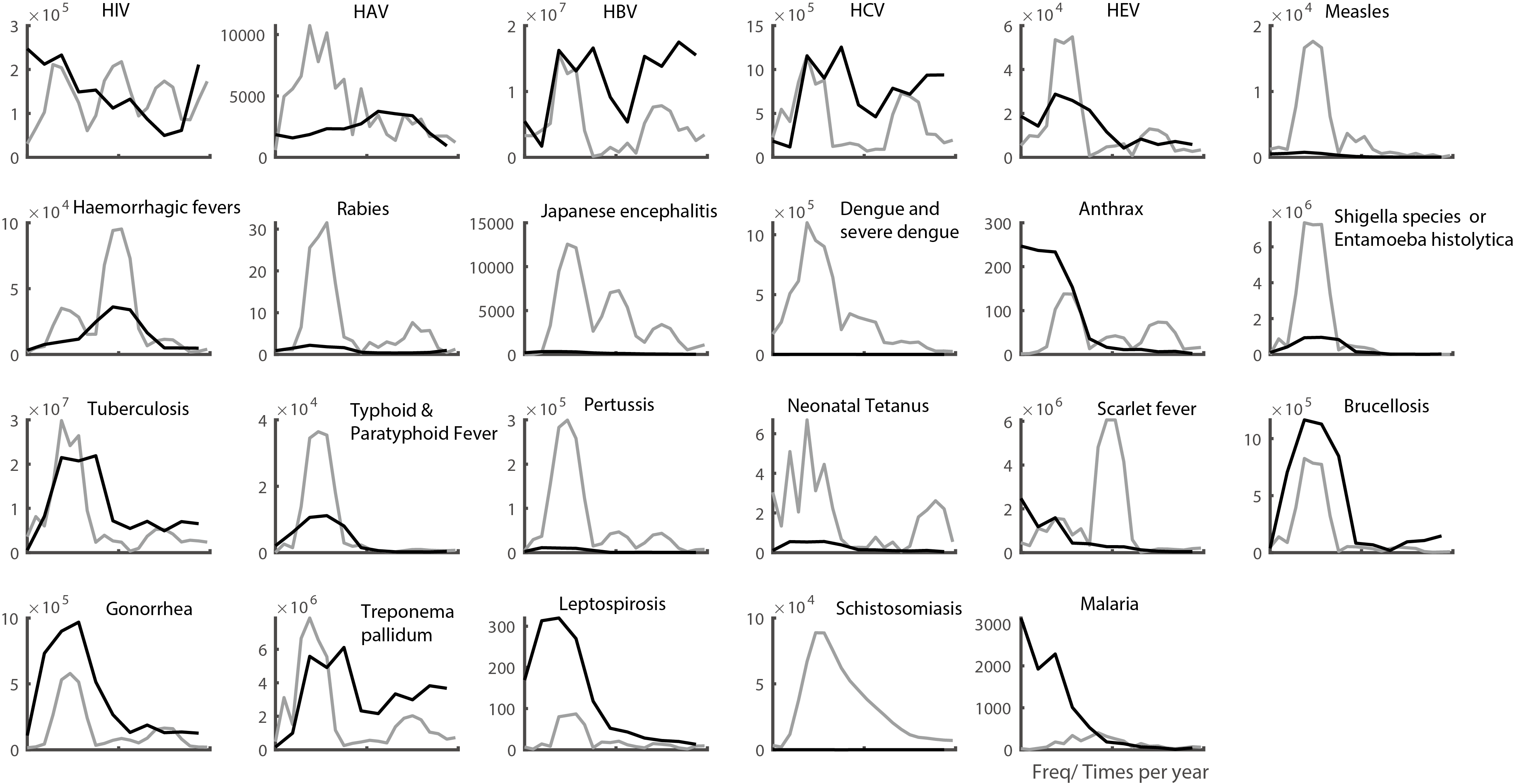

Supplement: Multimedia Appendix 5 [file publichealth_v8i6e35343_app5.png]
